# Secretome analysis of rice suspension-cultured cells infected by *Xanthomonas oryzae* pv*.oryza (*Xoo)

**DOI:** 10.1186/s12953-016-0091-z

**Published:** 2016-02-02

**Authors:** Xian Chen, Zhiping Deng, Chulang Yu, Chengqi Yan, Jianping Chen

**Affiliations:** College of Agriculture and Biotechnology, Zhejiang University, Hangzhou, China; State Key Laboratory Breeding Base for Zhejiang Sustainable Pest and Disease Control, MOA Key Laboratory of Biotechnology in Plant Protection, Zhejiang Provincial Key Laboratory of Plant Virology, Institute of Virology and Biotechnology, Zhejiang Academy of Agricultural Science, Hangzhou, 310021 China

**Keywords:** Bacterial blight, 2-D DIGE, MS, Secretome, Xoo3654

## Abstract

**Background:**

Rice bacterial blight (BB) caused by *Xanthomonas oryzae* pv*.oryzae (*Xoo) is one of the most devastating bacterial diseases in rice-growing regions worldwide. The rice-Xoo interaction is a classical model for studying the interaction between plants and pathogens. Secreted proteins play important roles in plant-bacterial interactions, but are poorly studied in the rice-Xoo system. Rice cv. Nipponbare is highly susceptible to Xoo. Here, we used two-dimensional difference gel electrophoresis (2D-DIGE) coupled with MALDI-TOF/TOF mass spectrometry (MS), to investigate secreted proteins in Nipponbare embryo cell suspension culture infected by Xoo.

**Results:**

A total of 32 protein spots changed significantly (*p* < 0.05) by more than 1.5 fold in gel intensity after Xoo inoculation, and were identified by MS. They represent protein products of 11 unique genes, seven from rice and four from Xoo. Of the rice proteins, six up-regulated proteins are involved in cell wall modification, the TCA cycle, glycolysis and redox, while a down-regulated protein, CHIT16, is involved in plant defense. Quantitative Real-Time PCR showed that transcript levels were not correlated with secreted protein levels. Of the Xoo proteins, three of them were possibly located in the extracellular space as shown by transient expression assays in rice protoplasts. Two of the Xoo proteins were previously reported to be likely involved in pathogenicity, and the third gene, Xoo3654, is likely a negative regulator of Xoo virulence as its overexpression reduced Xoo pathogenicity in our study.

**Conclusion:**

Among the secreted proteins that responded to Xoo inoculation, we identified rice proteins involved in cell defense and Xoo proteins involved in pathogenicity. Our study also showed that Xoo3654 (X2) protein is likely a novel negative regulator of Xoo virulence. These results not only help us better understand the interaction between susceptible rice and Xoo, but also serve as a reference for studying the interaction between other plants and their pathogens.

**Electronic supplementary material:**

The online version of this article (doi:10.1186/s12953-016-0091-z) contains supplementary material, which is available to authorized users.

## Background

Bacterial Blight (BB) caused by *Xanthomonas oryzae* pv*.oryza (*Xoo) is a vascular disease and one of the most serious diseases in rice. Xoo can invade rice xylem tissue either through wounds or stomata, causing systemic infection [1–3]. The most cost-effective way to control this disease is using resistant varieties [4]. A total of 37 resistance genes have been reported, 26 of which are dominant and the rest recessive [5]. Among these, six resistance genes have been cloned [6–11] and one of them, Xa21, is a NB-LRR protein kinase localized on the cell membrane that interacts with AvrXa21 and activates the rice immune system [9]. Due to the emergence of new physiological races of Xoo, many resistant varieties succumb to disease after a few years of cultivation [4, 12, 13]. This highlights the need to better understand the molecular basis of the complex interaction between susceptible rice and Xoo.

Secreted proteins play crucial roles in host - pathogen interactions [14, 15] and recent studies of these proteins by plant proteomics have led to a new field of study, the plant secretome [16]. The aim of plant secretome studies is to describe all the proteins secreted by a cell, tissue or organism at any given time or under certain conditions, and to understand the machineries for protein transport, protein interaction and protein modification [17]. The plant secretomes of *Arabidopsis thaliana* [18], maize [19], tobacco [20], medicago [21] and rice [22] have been studied using the in vitro cell suspension culture system. Secretome analysis of susceptible *A. thaliana* interacting with virulent bacteria has identified some extracellular host proteins lacking the traditional signal peptide [23]. In addition, a study of the rice plasma membrane identified proteins involved in early defense response to Xoo [24]. Proteomic studies of the Xoo secretome from its in vitro culture and in infected rice leaves showed that some components of the Xoo secretome were involved in its pathogenicity [25]. However, there have been no reports of changes in the rice secretome in response to Xoo infection in this study.

Here, secreted proteins were extracted from susceptible rice embryo suspension -cultured cells at 0 h (mock inoculation) and 24 h post-inoculation with Xoo strain PXO124(P10), and labeled with fluorescent dyes. Two-dimensional difference gel electrophoresis (2D-DIGE) was performed to separate proteins, Decyder 2D software was used to analyze the gel images, and MALDI- TOF/TOF mass spectrometry (MS) was used to identify the Xoo-responsive proteins. As a result, we discovered eight differentially expressed proteins from susceptible rice and four from Xoo.

## Results

**Fig. 1:**
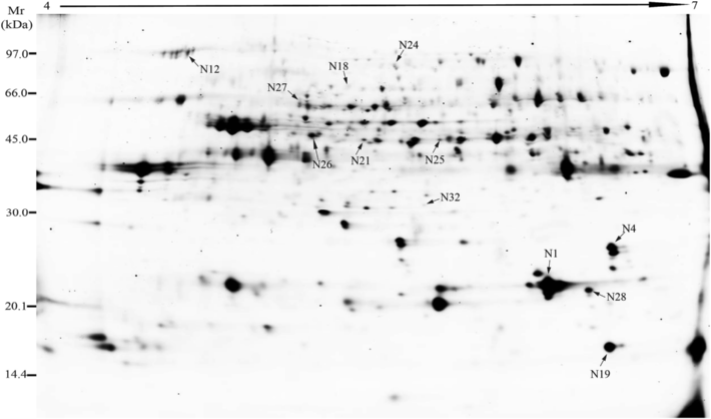
2D-DIGE analysis of secreted proteins from Nipponbare suspension-cultured cells 0 h and 24 h after inoculation with Xoo. Secreted protein (150 μg) from each sample were labeled with Cy3 and Cy5 respectively, loaded and separated on 24 cm strips (pH = 4–7) in the first dimension, followed by electrophoresis on 12.5 % SDS-PAGE gels in the second dimension. Gels were scanned with a DIGE scanner, merged and shown as a grayscale image. Protein spots responsive to Xoo-inoculation are marked by numbered arrows and identified in Tables 1 and 2. Relative mobility (Mr) markers were the protein ladder kit purchased from GE Healthcare (USA)

In preliminary experiments we tested three protein isolation approaches and found that the phenol-methanol method was most suitable for obtaining the secreted proteins for 2D-DIGE analysis [26, 27]. Then, we performed 2D-DIGE and MS to analyze the secreted proteins from mock and Xoo-infected rice Nipponbare suspension-cultured cells (Fig. 1). More than 500 protein spots were detected reproducibly on gels by the Decyder 2D software of which 32 changed in intensity significantly (*p* < 0.05) and by more than 1.5 fold when compared with the control. After analysis by MS, 32 proteins corresponding to 11 unique genes were matched to the NCBI database. Among those proteins, seven were from rice and four from Xoo.

Of the proteins identified from rice, six were up-regulated and maybe involved in glycolysis (2,3-bisphosphoglycerate-independent phosphoglycerate mutase, spot N18, Fig. 1), redox (plastocyanin-like domain containing protein, spots N19/N21; copper/zinc superoxide dismutase, spot N28), TCA cycle (succinyl-CoA ligase beta-chain, spot N26) and cell wall structure modification (beta-galactosidase, spot N24; cellulase, spot N27), respectively. The down-regulated protein is involved in defense (CHIT16, spot N32) (Table 1). In further analysis to predict their subcellular location, SignalP software predicted that the plastocyanin-like domain containing protein, beta-galactosidase, cellulase and CHIT16 contain signal sequences or cleavage sites. PRORT II prediction gave similar results to SignalP. Apart from succinyl-CoA ligase beta-chain and copper/zinc superoxide dismutase, the proteins were predicted to be extracellular or located in the plasma membrane (Table 1). These results help to confirm that our secreted-protein extraction method is suitable for 2D-DIGE analysis.

**Table 1:** The list of Xoo-responsive secreted rice proteins in rice suspension-culture medium identified by MS/MS. The average fold change of Xoo-inoculated to mock (positive numbers) or mock to Xoo-inoculated (negative numbers) was calculated from three biological replicates

No^a^	Accession No.	Locus	Match peptide^b^	SC %^c^	Average fold change^d^	Molecular function and or property	Cell location	SignalP^e^
N18	gi|115464537	Os05g0482700	12	31	9.85 ± 0.047	2,3-bisphosphoglycerate-independent phosphoglycerate mutase	extracellular	-
N19	gi|125560875	OsI_28556	5	34	8.19 ± 0.016	plastocyanin-like domain containing protein, putative, expressed	extracellular	Yes
N21	gi|125560875	OsI_28556	5	21	7.36 ± 0.024	plastocyanin-like domain containing protein, putative, expressed	extracellular	Yes
N24	gi|222618730	OsJ_02342	13	15	5.2 ± 0.024	beta-galactosidase	extracellular	Yes
N26	gi|115447367	Os02g0621700	25	66	4.53 ± 0.045	succinyl-CoA ligase beta-chain, mitochondrial precursor, putative, expressed	mitochondrial	-
N28	gi|115473931	Os07g0665200	8	64	1.63 ± 0.043	copper/zinc superoxide dismutase, putative, expressed	cytoplasmic	-
N27	gi|115481730	Os10g0370500	16	38	2.65 ± 0.024	cellulase, putative, expressed	plasma membrane	Yes
N32	gi|115450541	Os03g0132900	13	53	−5.83 ± 0.024	CHIT16 - family protein precursor, expressed	extracellular	Yes

^a^spot number as given in Fig. 1. Some proteins from different spots correspond to the same gene suggesting they may have been post-translationally modified

^b^number of matched peptides

^c^sequence coverage

^d^Fold change with *p* <0.05

SignalP^e^ “Yes” means protein was predicted to have a signal peptide

**Fig. 2:**
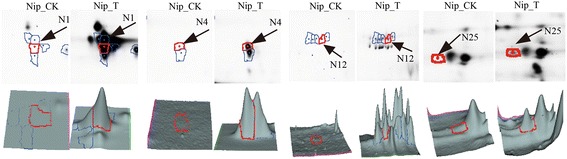
Xoo proteins in the secreted protein fraction identified by 2-D DIGE. Zoom-in images (top panel) of each spot from mock (CK) and Xoo-infected (T) samples are shown, respectively. These bacterial blight proteins (Xoo3479, Xoo3654, Xoo0842 and Lipay) were detected only from inoculated cells and were not present in mock cells. CK: Secreted proteins from nip suspension- cultured cells; T: Secreted proteins from nip suspension-cultured cells post-inoculation with Xoo

Nineteen differentially expressed protein spots corresponding to four unique proteins from Xoo (Fig. 2) were identified by BLAST in the Prosite, Pfam and InterPro databases and their predicted functions or properties are shown in Table 2. One is involved in degrading the rice cell wall (LipA, spot N25) and one in transport (transporter, spot N12), but the functions of the others are unclear. All are predicted to be located in the extracellular space by PSORT, and only one (Xoo3479, spot N1) has no signal peptide when analyzed in SignalP 4.1. Xoo3479 was not predicted to have a signal peptide when assessed with SecretomeP, and it is therefore possible that this protein was transported to the extracellular space through a non-classical pathway.

**Table 2:** The list of secreted Xoo proteins in rice suspension-culture medium identified by MS/MS

No^a^	Accession No.	Protein name	Match peptide^b^	SC %^c^	Average fold change^d^	Molecular function	Cell location	SignalP
N1	gi|58583102	Hypothetical protein Xoo3479	12	95	157 ± 0.013	Unclear	extracellular	-
N4	gi|84625311	hypothetical protein Xoo3654	18	68	56.99 ± 0.027	Unclear	extracellular	Yes
N12	gi|58580465	hypothetical protein Xoo0842	20	30	14.23 ± 0.023	Pathogenicity	extracellular	-
N25	gi|256032659	Lipay	10	46	4.92 ± 0.024	Pathogenicity	extracellular	-

^a^spot number as given in Fig. 1

^b^number of matched peptides

^c^sequence coverage

^d^Fold change with *p* <0.05

**Fig. 3:**
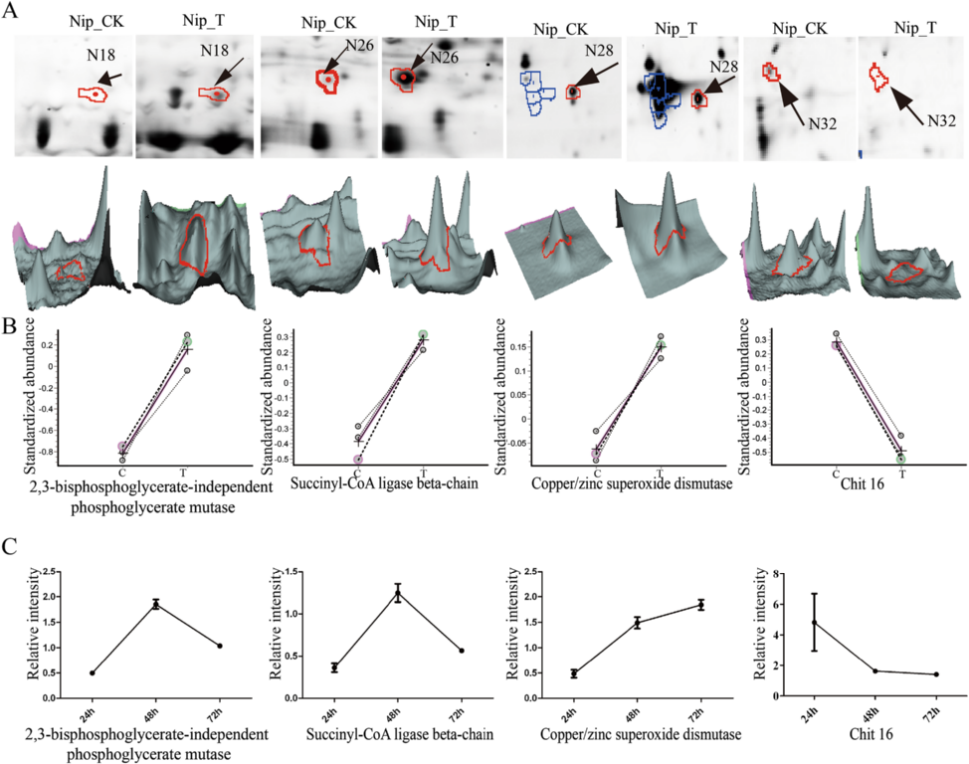
Xoo-responsive rice proteins identified by 2-D DIGE. **a**, zoom-in images (top panel) and three-dimensional view (bottom panel) of each spot from Nipponbare mock (Nip-CK) and Xoo-infected (Nip-T) samples are shown; **b**, Changes in protein abundance of the four rice proteins in the secreted protein fraction of suspension-cultured cells after 24 h inoculation. **c**, the mRNA expression of the four rice genes in leaves of rice seedlings 24, 48 and 72 h after inoculation by Xoo

To investigate the relationship between the identified proteins and their transcription, real time RT-PCR was performed to analyze the transcriptional activities of four randomly selected rice genes corresponding to the rice proteins identified. Samples were tested at 0 h, 24 h, 48 h, and 72 h after Xoo- inoculation. There was a poor correlation between changes in mRNAs and the corresponding proteins (Fig. 3). For example, 2,3-bisphosphoglycerate-independent phosphoglycerate mutase (Spot N18) and succinyl-CoA ligase (Spot N26) were up-regulated, whereas their transcripts were down-regulated at 24 h and up-regulated at 48 h. The transcript of the up-regulated copper/zinc superoxide dismutase was also down-regulated at 24 h. The mRNA transcriptional level does not always correlate well with the protein expression levels [28]. For example, the cold-stress response protein, 2,3-bisphosphoglycerate-independent phosphohlycerphate mutase, was up-regulated, but transcription was down-regulated [29] similar to our results. The transcription of many PR proteins was first up and then down when treated with Jasmonate (JA) [30]. It should be noted that seedling leaves were used to measure transcript levels, while the secreted protein levels were determined from suspension cultured cells. And gene expression in infected leaves may also influenced by the circadian rhythm.

**Fig. 4:**
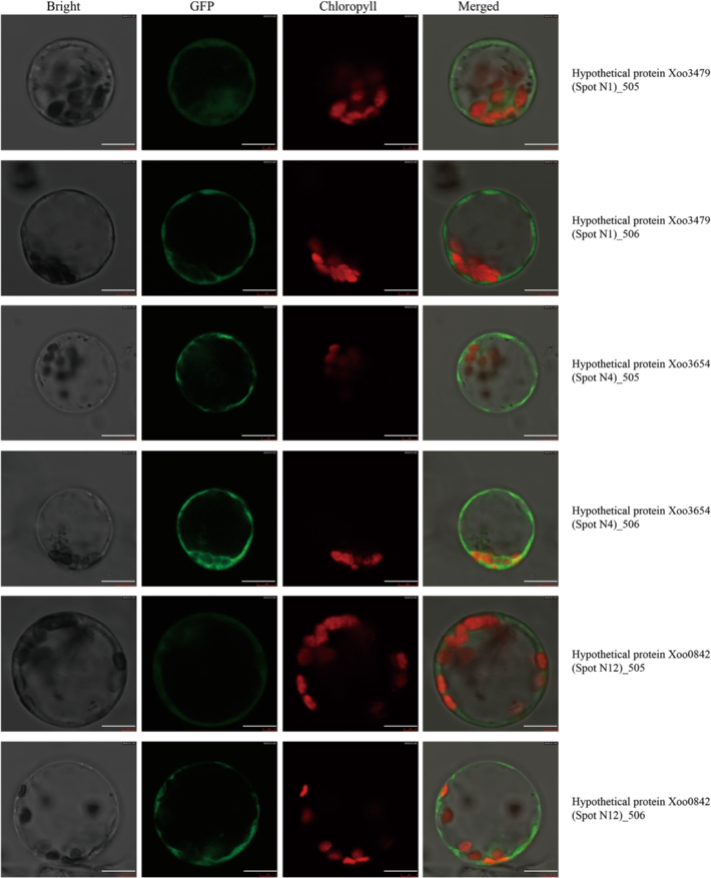
Subcellular location of three identified Xoo secreted proteins in rice protoplast cells. To exclude interference of the GFP protein with the location of the tested genes, bacterial blight genes were cloned into the pGW505 (_505, 35S: Gene-GFP fusion) and pGW506 (_506, 35S: GFP- Gene fusion) vectors. GFP signals were detected 16 h after transient expression in rice protoplasts. Images of rice protoplasts observed under bright light, GFP, chlorophyll channels, and false-colour signal images are shown in panels from left to right, respectively. Superimposed images are shown in the rightmost panel. GFP protein fusions of Xoo3479, Xoo3654 and Xoo0842 were all observed on plasma membranes. Bar = 10 μm

The N-and C-termini of three candidate Xoo genes were fused with enhanced green fluorescent protein (GFP) and the 35S::GFP plasmids were transfected into rice protoplasst, repectively. The GFP fluorescent signals of Xoo0842, Xoo3479 and Xoo3654 were detected only in the plasma membrane of rice protoplasts, confirming their extracellular location (Fig. 4). This suggests that those Xoo proteins can be secreted into the extracellular space after expression in rice protoplasts, but further studies are needed to determine whether they are effectors or elicitors in pathogenicity.

**Fig. 5:**
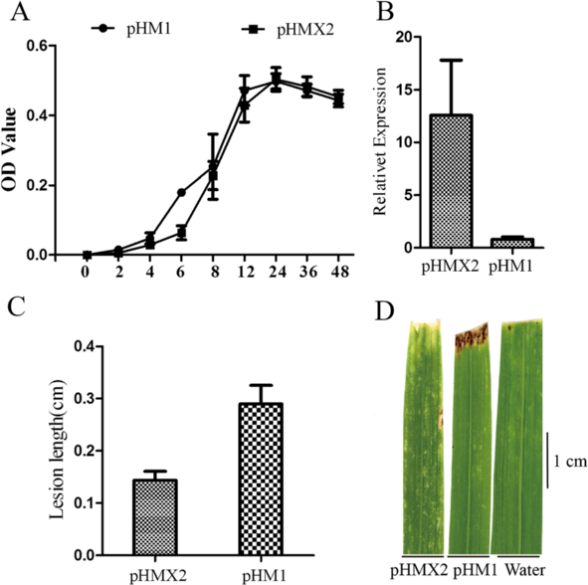
Over-expression of Xoo_3654 (X2) in PXO124 and its effects on pathogenicity. **a**: Growth curve of construct strains pHMX2- PXO124 and pHM1-PXO124. Compared with mock plasmid pHM1, X2 slightly reduced the growth rate. **b**: Expression of X2 gene in construct strains pHMX2- PXO124 and pHM1-PXO124. Total RNA was extracted from pHM1-PXO124 and pHMX2-PXO124, and 500 ng RNA was reverse transcribed by the iScript cDNA synthesis kit (Bio-RAD) and quantified using qRT-PCR. 16 s-rDNA was used to normalize total RNA amounts. Expression levels of X2 in pHMX2-PXO124 are more than fifteen times higher than in pHM1-PXO124. **c**: Pathogenicity test showing reduction of lesion length in young leaves inoculated with pHMX2- PXO124 compared to those inoculated with pHM1-PXO124. **d**: Phenotypes of lesions in 21-dyas rice leaves inoculated with pHMX2- PXO124 and pHM1-PXO124 for 14 days

To characterize the function of Xoo*-*3654 (X2) gene in pathogenicity, we cloned it into Xoo strain PXO124 (pHMX2-PXO124 ). Growth curve analysis showed that X2 slightly reduced the growth rate (Fig. 5a). qRT-PCR showed that the expression level of X2 in pHMX2-PXO124 was over fifteen times higher than in pHM1-PXO124 (Fig. 5b). In a virulence assay using the leaf clipping method (Fig. 5c & d), pHMX2- PXO124 was less virulent than pHM1-PXO124, with shorter lesion length on leaves of adult rice (wild type). These results suggest that Xoo3654 acts as a negative regulator of this pathovar of Xoo.

## Discussion

Secreted proteins play crucial roles in a number of physiological and pathological processes, such as growth and development [31], cell division and differentiation [32], defense- and stress-related responses [33]. Suspension-culture has been a preferred and widely used system for secretome analysis in plants and other organisms, including mammals [34], bacteria [25, 35] and fungi [36]. In this study, we used 2D-DIGE to analyze the secreted proteins in a rice embryo cell suspension culture challenged by Xoo.

Plants have evolved defense mechanisms to protect themselves from biotic and abiotic stresses, one of the most important of which involves Pathogenesis-Related proteins (PR proteins). Many chitinases are PRs, and can be induced by fungi [37], bacteria and viruses [38]. Chitinases are not only involved in plant growth and development [39], but also enhance plant defense [40]. Overexpression of chitinase can enhance defense against fungi in transgenic plants [41]. In this study, CHIT16 (Spot N32) is a chitinase belonging to the third group of the chitinase family. It contains a signal peptide and a transmembrane domain, which suggests that it is a secreted protein. However, this protein was down-regulated in response to Xoo infection. Plant susceptibility to pathogens is very complex, and may include non-recognition of the pathogen or inability to activate the defense system. Whether the down regulation of CHIT16 is the main cause of susceptibility of Nipponbare to Xoo needs to be further investigated. The structure of the cell wall can be modified in response to developmental or environmental stress [42]. Cellulose is the main structural component of the primary cell wall and is therefore one of the most important compounds in the first line of physical defense in green plants. Cellulases are enzymes that degrade cellulose into glucose. Although they are involved in cell growth and proliferation, they also have a destructive effect on cells [43]. Pathogen cellulases may also be associated with their pathogenicity [44]. Here, we observed one cellulase (Spot N27, Uniprot ID Q8RU06 ) that was up- regulated under the stress of Xoo infection. This cellulase may be involved in degrading the rice cells walls. It needs to be confirmed whether this response made the cells conducive to Xoo infection and so contributed to reduced host resistance.

Several proteins secreted from Xoo were identified in the culture medium, contrasting with results from a suspension culture of resistant rice cells (unpublished data). Although the pathogenicity of Xoo is quite complex, it is also genetically determined and observes the rules of “Special and Niche Characteristics” [45]. Aparna [46] found that Lipay (Spot N25) was an esterase, which is not only involved in degrading the rice cell wall, but also plays a role as a secretory virulence factor eliciting the innate immune response. There are no published studies on the other secreted proteins we identified (Xoo0842, Xoo3479 and Xoo3654). Bioinformatics analysis of Xoo0842 (Spots N12) in the NCBI database, suggests that it has six Lbr-YadA domains, one ESPR domain and two Hia domains. This suggests that Xoo0842 meybe can transported to the extracellular space through the type V secretory pathway, and may be a transport protein having the similar function of virulence to that of YadA. In the recent study of Wang et al. [25], Xoo0842 was only observed in infected leaves and not in vitro culture medium. We deduce that this protein is only expressed in infection of rice and may have some function of pathogenicty of PXO124. This still needs further validation. In Wang’s study*,* Xoo3479 was detected in both the in vitro medium and, at higher levels, in infected leaves [25]. We also observed Xoo3479 (Spots N1) in Nipponbare suspension-cultured medium. Xoo3654 (Spot N4) is a novel secreted protein. Overexpression of Xoo3654 slightly reduced the pathogenicity of PXO124, suggesting its role as a negative regulator in bacterial virulence, although the detailed molecular mechanisms needs further investigation.

## Conclusions

The rice - Xoo interaction is a classical model for studying plant - pathogen interactions. Here, we first use 2D-DIGE to analyze differentially expressed secreted proteins in susceptible rice suspension-cultured cells incubated with Xoo. The identified proteins are involved in various biological processes, including defense, cell wall modification, redox, glycolysis and the TCA cycle. In addition, four Xoo secreted proteins were also observed in this study. Subcellular location showed that three of these proteins were located in the extracellular region. Meanwhile, Xoo3654(X2) was shown to affect Xoo virulence as its overexpression leads to decreased pathogenicity. These results not only help us better understand the interaction between susceptible rice and Xoo, but also serve as a reference for studying the interaction between other plants and pathogens.

## Methods

### Plant and bacterial material

Mature rice seeds (*O. sativa* subsp. *japonica* var. Nipponbare) were dehulled and sterilized in 70 % ethanol for 2 min and then in 20 % sodium hypochlorite for another 30 min followed by extensive washing in distilled water to remove the disinfectant. Sterilized seeds were placed on N6 callus induction medium [47] at 28 °C under a 16/8 h light/dark photoperiod regime for one month to induce calli. Growing calli (0.5–1.0 g) were transferred into liquid N6 medium and shaken at 150 rpm in the dark. The suspension culture was sub-cultured weekly until the cells appeared dense, uniform and light yellow. Xoo strain P10(PXO124) was cultured on PSA liquid medium (1 % w/v peptone, 1 % w/v sucrose) at 28 °C for 48 h and adjusted to 10^8^ CFU ml^−1^ before inoculation to the rice suspension culture 3 days after sub-culturing. For pathogenicity assay, leaves from 21-d rice seedlings were inoculated with Xoo(OD = ~0.8) using leaf-cutting method [48].

### Preparation of secreted proteins from rice suspension-cultured cells

After sub-culturing for three days, the rice culture medium was harvested 0 h and 24 h after Xoo inoculation. Calli were removed by a nylon filter (0.18 mm). The filtered medium was centrifuged at 20 000 × g for 20 min and the clear supernatant was freeze-dried. Total protein was extracted using a modified phenol-methanol method [26, 27]. At least three biological replicates were prepared. The secreted proteins were further purified using the 2-D Clean Up Kit (GE healthcare, USA) and their concentrations determined using a 2-D Quant kit (GE healthcare, USA).

### 2D-DIGE, image scanning and analysis

Prepared secreted proteins were separated by 2D-DIGE as described previously [49]. The Cy2, Cy3, Cy5 labeled samples (50 μg) were mixed and loaded on the strips (linear, 24 cm, pI 4–7, GE Healthcare, USA) for the first dimension separation. Next, the strips were placed on top of 12.5 % SDS-PAGE gels for the second dimension electrophoresis. Protein spots on gels were scanned using an Ettan DIGE Scanner (GE Healthcare, USA) and the images were analyzed using Decyder 2D software (Version 7.0, GE Healthcare, USA). Finally, spots from different gels were matched using Biological Variation Analysis. Only spots present in all gels and which exhibited statistically significant changes in intensity (≥1.5 fold or ≤ −1.5 fold, *p* <0.05) were considered to be differentially expressed proteins.

### In gel digestion and MS analysis

About 500 μg secreted proteins were loaded on the strips, separated by 2-DE and stained with Coomasie Brilliant Blue (CBB) R-250. Differentially expressed protein spots were manually excised from the stained 2-D gel and transferred to a sterile tube (1.5 ml) with 30 % (w/v) Acetonitrile (ACN) and NH_4_HCO_3_ (100 mmol) solution to remove the CBB stain. After vacuum drying, the spots were digested in 30 μl enzyme buffer (50 mmol NH_4_HCO_3_, 50 ng/μl trypsin (Sigma, USA)) at 37 °C overnight. Then, the small peptides were back extracted using 60 % (w/v) ACN (containing 0.5 % w/v trifluoroacetic acid (TFA)) and dried under a steam of nitrogen. Finally, the peptide samples were re-suspended in 0.8 μl of 50 % (w/v) ACN (containing 0.1 % w/v TFA and 5 mg/ml αcyano-4-hydroxycinnamic acid(CHCA)) and analyzed using a ABI4700 MALDI-TOF/TOF mass spectrometer (Applied Biosystems, USA). All MALDI-TOF spectra were searched against the National Center for Biotechnology Information non-redundant (NCBInr) database using the GPS Explorer™ software (v3.6, Applied Biosystems) and MASCOT search program (v2.1 Matrix Science). Finally, based on the MALDI-TOF-MS, only protein scores > 95 (*p* <0.05) were accepted for the identification of protein samples.

### Bioinformatics analysis

Homologues of the identified proteins were searched in the RAP-DB (http://rapdb.dna.affrc.go.jp/) and RAGP (http://rice.plantbiology.msu.edu/) databases for matching against the NCBI database. Then, UniProt (http://www.uniprot.org/) and Pfam (http://www.sanger.ac.uk/) databases were used to determine their functions. In addition, SignalP (version 4.1, http://www.cbs.dtu.dk/services/SignalP/) and SecretomeP (version 2.0, http://www.cbs.dtu.dk/services/SecretomeP/) were used to predict their secretion pathways and PSORT II (http://psort.hgc.jp/form2.html) to predict their subcellular location.

### RNA extraction and quantitative real time PCR for gene expression

Primers were designed according to the gene sequences in the RAP-DB and RAGP databases, using Primer Premier 5.0 (Additional file 1: Table S1). Nipponbare seedlings were cultivated on greenhouse until the four to five-leave stage. The rice leaves were inoculated with Xoo at 0 h, 24 h, 48 h and72h post infection by leaf-cutting method [48]. Total RNA was extracted from infected rice leaves using TRIzol reagent (Invitrogen, Germany). Residual DNA was removed by DNase I RNase free (TaKaRa, Japan) and the first strand cDNA was synthesized using the Synthesis Kit for RT-qPCR (Bio–RAD, USA) following the manufacturer’s instructions. Real time PCR with SYBR Green Real time PCR Master Mix (TOYOBO, Japan) was performed on a 7900HT Fast Real time PCER system (Life Technologies, USA), and PCR conditions were as follows: 94 °C for 3 min, then 45 cycles of 95 °C for 30s, 58 °C for 45 s and 72 °C for 45 s. *Actin* (AK060893) was used as a reference gene, and the relative gene expression was calculated using the 2^-ΔΔct^ method. All experiments were performed in triplicate with the cDNA prepared from different samples.

### Vector construction and subcellular localization of Xoo identified proteins

The cDNAs of Xoo0842, Xoo3479 and Xoo3654 were amplified using their respective primers (Additional file 1: Table S2), and the full-length coding regions were fused in-frame with GFP in pGW505 and pGW506 using the Gateway technology (Invitrogen, USA) followed by transient expression in rice protoplast cells, using the rice protoplast gene expression system and the PEG method [49]. The GFP signal was excited at 395 nm and observed at 450–490 nm using a confocal laser scanning microscope (Olympus, Japan). Chlorophyll signals were excited at 436 nm and observed at 500–530 nm.

### Over-expression of Xoo3654 in PXO124 and growth assay analysis

To investigate whether Xoo3654 affects pathogenicity, we cloned its coding region and inserted it into pHM1, a expressing vector with the constitutive lac promoter [50]. The resultant vector pHMXoo3654 (pHMX2) was verified by sequencing (Biosune, Hangzhou, China). Purified pHMX2 and pHM1 (mock) plasmids were electroporated into PXO124(P10) competent cells. The transformed cells were selected on NA medium (1 % tryptone, 0.1 % yeast extract, 1 % sucrose, 0.3 % peptone, 1.5 % agar) containing spectinomycin(25 μg · mL^−1^) at 28 °C for 4 d, and were subsequently verified by sequencing (Biosune, Hangzhou, China). For the growth assay, the colonies of Xoo strains were grown in NB medium (NA medium without agar) for 24 h. Then, 100 μl of culture medium of each strain adjusted to 0.5 × 10^8^ colony formation units (CFU) per ml, was inoculated into fresh NA liquid medium and shaken at 200 rpm at 28 °C. Three replicate samples were collected at each indicated time point (up to 48 h), and their optical densities were measured at 600 nm (OD_600_).

### RT-PCR for X2 construct and its effect on pathogenicity

Total RNA extraction from construct strains and quantitative real time PCR for Xoo3654 were performed using the methods described above for Nipponbare. The 16 s rDNA was used to normalize total RNA amount. For pathogenicity tests, constructs Xoo (OD_600_ = 0.8) were incubated into 45-day rice using the leaf-cutting method, and NA medium was used as a control. The infected plants were grown in a green house at 25–30 °C with a 12 h photoperiod and 60 % relative humidity. The lesion length was measured 14 days later. Each experiment was conducted three times.

## Additional files

Additional file 1: Table S1. Primer sequences used for quantitative RT-PCR of differentially expressed genes in susceptible rice subject to Xoo inoculation. (DOCX 14.3 kb) Additional file 2: Table S2. Primer sequences used for transient expression of Xoo genes in rice protoplast cells. (DOCX 13.8 kb) Additional file 3: Table S3. Primer sequences used for over-expression and RT-PCR of Xoo3654 gene. (DOCX 14.1 kb)

## Abbreviations

2-D DIGE: two-dimensional difference in gel electrophoresis
ACN: Acetonitrile
BB: bacterial blight
CHCA: α- cyano −4-hydroxycinnamic acid
CHIT 16: chitinase 16
MS: mass spectrometry
NB-LRR: nucleotide-binding site leucine-rich repeat
TCA: tricarboxylic acid cycle
TFA: trifluoroacetic acid
