# Supplementary material for: Secretome analysis of rice suspension-cultured cells infected by Xanthomonas oryzae pv.oryza (Xoo)
Source: Proteome Sci. 2016 Feb 2;14:2. doi: 10.1186/s12953-016-0091-z (PMC4735954; doi:10.1186/s12953-016-0091-z)
Supplement: Additional file 1: Table S1. — Primer sequences used for quantitative RT-PCR of differentially expressed genes in susceptible rice subject to Xoo inoculation. (DOCX 14.3 kb) [file 12953_2016_91_MOESM1_ESM.docx]

**Supplementary Table.1** Primer sequences used for quantitative RT-PCR of differentially expressed genes in susceptible rice subject to Xoo inoculation

| **Spot No** | **NCBI Accession number** | **Name** | **Sequence (5’-3’)** | **Product size (bp)** |
| --- | --- | --- | --- | --- |
| N18 | gi\|115464537 | 2,3-bisphosphoglycerate-independent phosphoglycerate mutase | F: CACCACCAGAGATTGAGAG | 255 |
|  |  |  | R: ACTTGCCACTAAGGATAGC |  |
| N26 | gi\|115447367 | succinyl-CoA ligase | F: GGCAATGGCTACAATGGA | 235 |
|  |  |  | R: AACAACAGGCACCTTCAG |  |
| N28 | gi\|115473931 | copper/zinc superoxide dismutase | F: GACCACACTTCAATCCTACT | 265 |
|  |  |  | R: ATGATTCCGCAAGCAACT |  |
| N32 | gi\|115450541 | CHIT16 | F: GCCACCAACATCATCAAC | 130 |
|  |  |  | R: AGGCTATGTTATCTCCGTATC |  |
