# Supplementary material for: Secretome analysis of rice suspension-cultured cells infected by Xanthomonas oryzae pv.oryza (Xoo)
Source: Proteome Sci. 2016 Feb 2;14:2. doi: 10.1186/s12953-016-0091-z (PMC4735954; doi:10.1186/s12953-016-0091-z)
Supplement: Additional file 2: Table S2. — Primer sequences used for transient expression of Xoo genes in rice protoplast cells. (DOCX 13.8 kb) [file 12953_2016_91_MOESM2_ESM.docx]

**Supplementary Table.2** Primer sequences used for transient expression of Xoo genes in rice protoplast cells.

| **Spot No** | **NCBI Accession number** | **Name** | **Sequence (5’-3’)** | **Product size (bp)** |
| --- | --- | --- | --- | --- |
| N1 | gi\|58583102 | Xoo3479 | F: TATG CAG CAC GCG TTT CTT TCT | 497 |
|  |  |  | R: TTCA AAC GGT GTA GTT GGG AAT |  |
| N4 | gi\|84625311 | Xoo3654 | F: TATG AAG CTT GGA ATG ACG CAT | 566 |
|  |  |  | R: TTCA GCA GCC GCT CAT GCA AAT |  |
| N12 | gi\|58580465 | Xoo0842 | F: TATG AAT CAG ATT TAT CGC AAG | 3799 |
|  |  |  | R: TTTA CCA GCC GAA ACC GGC ACC |  |
