# Supplementary material for: Secretome analysis of rice suspension-cultured cells infected by Xanthomonas oryzae pv.oryza (Xoo)
Source: Proteome Sci. 2016 Feb 2;14:2. doi: 10.1186/s12953-016-0091-z (PMC4735954; doi:10.1186/s12953-016-0091-z)
Supplement: Additional file 3: Table S3. — Primer sequences used for over-expression and RT-PCR of Xoo3654 gene. (DOCX 14.1 kb) [file 12953_2016_91_MOESM3_ESM.docx]

**Supplementary Table.3** Primer sequences used for over-expression and RT-PCR of Xoo3654 gene.

| **No** | **NCBI Accession number** | **Name** | **Sequence (5’-3’)** | **Product size (bp)** |
| --- | --- | --- | --- | --- |
| X2 | gi\|84625311 | Xoo3654 | F: GTCGACATGAAGCTTGGAATGACGCAT | 566 |
|  |  |  | R: GGTACCTCAGCAGCCGCTCATGCAAAT |  |
| X2 | gi\|84625311 | Xoo3654 | RT-F: CCGATGCCTCCAGTCCGATT | 156 |
|  |  |  | RT-R: ACAGCGACAGTAGCCGATACG |  |
| CK | gi\|833360487 | 16s-  rDNA | RT_F：GACTTGGCTTCGCGATACAGG | 237 |
|  |  |  | RT-R：GGTCAAGATTCGACTGACCCG |  |
